# Advanced Modified Polyacrylonitrile Membrane with Enhanced Adsorption Property for Heavy Metal Ions

**DOI:** 10.1038/s41598-018-19597-3

**Published:** 2018-01-19

**Authors:** Xinfeng Zhang, Shujing Yang, Bing Yu, Qinglong Tan, Xiaoyan Zhang, Hailin Cong

**Affiliations:** 1 Institute of Biomedical Materials and Engineering, College of Chemistry and Chemical Engineering, Qingdao University, Qingdao, 266071 China; 2 Laboratory for New Fiber Materials and Modern Textile, Growing Base for State Key Laboratory, College of Materials Science and Engineering, Qingdao University, Qingdao, 266071 China

## Abstract

Advanced modified polyacrylonitrile (PAN) membrane with high adsorption property for heavy metal ions was designed and fabricated for the first time. The introduced diazoresin-ethylenediaminetetraacetic acid (DR-EDTA) layer could effectively absorb the metal ion, such as Cu^2+^, Pb^2+^, Hg^2+^ in the waste water. The effects of layers, metal ion concentration, pH, temperature and cycle time were investigated. The results showed that the adsorption isotherms for Cu^2+^ were well fitted by Langmuir model. The maximum adsorption capacity of the modified membrane for Cu^2+^ was approximately 47.6 mg/g. In addition, the prepared PAN-(DR-EDTA)_3_ membrane could be regenerated more than 720 h based on their adsorption/desorption cycles. The results demonstrated that the modified PAN membrane could be used as effective adsorbents for heavy metal removal from waste water.

## Introduction

Nowadays, the industrial treatments of wastewaters flow into our living environment, which is non-biodegradable and carcinogenic^1^. It has been regarded as a serious environmental concern which can cause all sorts of diseases^2–5^. The trace heavy metal ions existed in raw wastewater which outflow from chemical laboratory, steam-electric power plants, metallurgy, mineral processing, nuclear fuel industry and so on^6,7^. The heavy metal ions generally exist in acidic solutions which is difficult to separate from the waste water^8^. Various methods have been developed to remove the heavy metal ions, including adsorption, filtration^9^, membrane separation^10^, ion exchange^11^, and so on. The adsorption is commonly used method due to its economic viability, simplicity and environmental protection^12,13^. Adsorption materials reported were not highly efficient and expensive and required to be used in severe or aggressive conditions^14^. Therefore, challenges still lie in the design of the polymer membrane structure.

Several adsorbents have been reported such as activated carbon^15^ and graphene oxide^16^. However, the adsorbents had some drawbacks such as high attrition rate, costly regeneration. And the limited sorption capacity of the adsorbents still limited in their practical applications to remove heavy mental ions^16^. Marta Izquierdo *et al*. investigated the performance of Posidonia oceanica for Cu^2+^ biosorption in the presence of EDTA. The presence of EDTA in the wastewater decreased the Cu^2+ ^^17^.

Recently, the PAN fiber membranes have been applied in many aspects, such as microfiltration^18^, ultrafiltration^19–21^, desalination^22–24^. However, the pure PAN fiber membrane could not absorb metal ions effectively, which restricted their further application in water treatment. To address this challenge, many attempts were performed on the surface modification of pure PAN membrane. For example, Sheng *et al*.^25^ thio-functionalized the surface of polyacrylonitrile with the selective and enhanced adsorption for Hg^2+^ and Cd^2+^. However, the adsorption for other heavy metal ions was unsatisfactory. Gang *et al*.^26^ prepared a highly selective and efficient chelating fiber with the surface modified by bis(2-pyridylmethyl)amino group. The prepared membrane could be easily recycled or reused toward the metal ions. Pimolpun *et al*.^27^ used NaOH ethanolic/aqueous solution to modify the surface of PAN fiber to introduce imine conjugated sequences, which could chelate metal ions. Unfortunately, the preparation processes of membranes were complicated and costly. In addition, although these membranes could absorb the metal ions to some degree, enhanced adsorption capacity of the membrane was limited as for the number of grafted functional absorb groups in the polymer chain.

**Figure 1:**
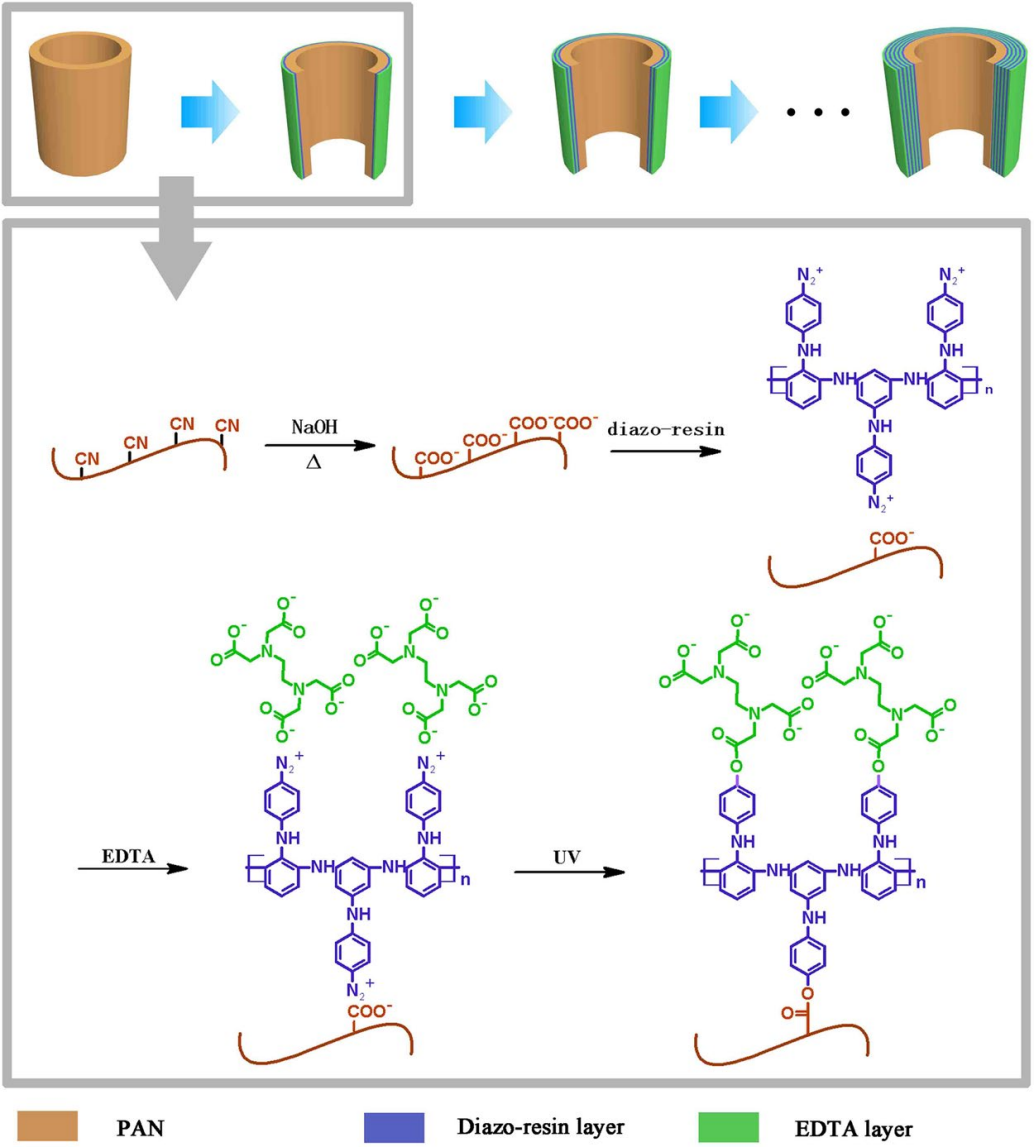
Scheme of grafting DR and EDTA onto the PAN membrane surface.

Herein, we introduced DR-EDTA layers on the surface of the pure PAN fiber membrane through layer-by-layer method. As shown in Fig. 1, the diazo resin with diazo groups could adsorbed the –COOH groups in PAN and EDTA by electrostatic interaction. Photo-crosslinking reaction occurred between diazo groups and–COOH groups after exposure processing. The EDTA could absorb metal ions and water simultaneously owning to the chelate ability and hydrophilic property. More importantly, membrane absorbance capacity toward metal ion could be controlled via DR-EDTA layers. Besides, the modified PAN membrane could absorb different heavy metal ions selectively. In this work, a novel kind of PAN-based modification membrane was prepared. The structure of PAN fiber membrane was characterized by FTIR, SEM and AFM. The adsorption performance of the as-prepared membrane, including the modification layer number effect, temperature effect, recycling properties as well as the water flux were thoroughly investigated.

## Results and Discussion

**Figure 2:**
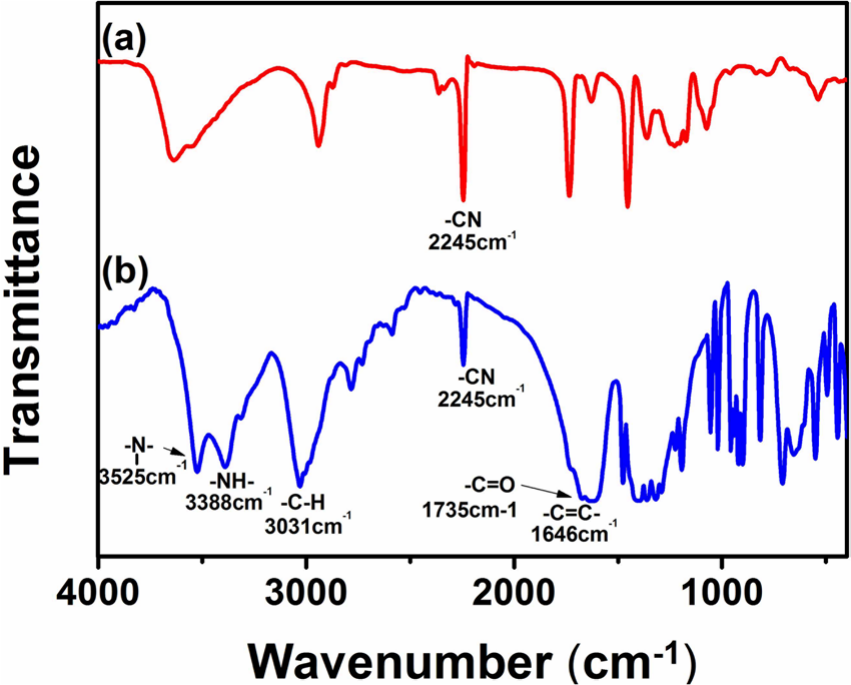
Infrared spectrum of (**a**) PAN-(DR-EDTA)0 and (**b**) PAN-(DR-EDTA)_3_.

The surface modification of PAN fiber with DR-EDTA layers were successfully performed by method of layer-by-layer self-assembly method. The structure of the modified PAN fiber membrane was analysised by FITR. As shown in Fig. 2, the characteristic peak of PAN fiber membrane at 2245 cm^−1^ and 1735 cm^−1^ could be caused by stretching modes of –CN and methyl acrylate units, respectively. After reaction, the adsorption band of The PAN cross-linked with DR and EDTA layers at 2245 cm^−1^ was remarkably reduced. This indicated that not all the –CN groups participate in the hydrolysis, which implied modification mainly occurred on surface of PAN fiber membrane. The stretching band of benzene skeleton in DR was observed at 1646 cm^−1^ which confirmed successful graft of DR layer^28–30^. Newly appeared peak at 3525 cm^−1^ were assigned to the combination of –OH and tertiary amino group. Additionally, the peak at 1735 cm^−1^ corresponded to the stretching vibration of –COO^−^ was observed which suggested that photo-crosslinking was occurred between PAN and DR, DR and EDTA, respectively. The peak between 3500–4000 cm^−1^ attributed to water. The water was removed after UV exposure. It was confirmed that EDTA layers were grafted on the surface of PAN fiber membrane.

**Figure 3:**
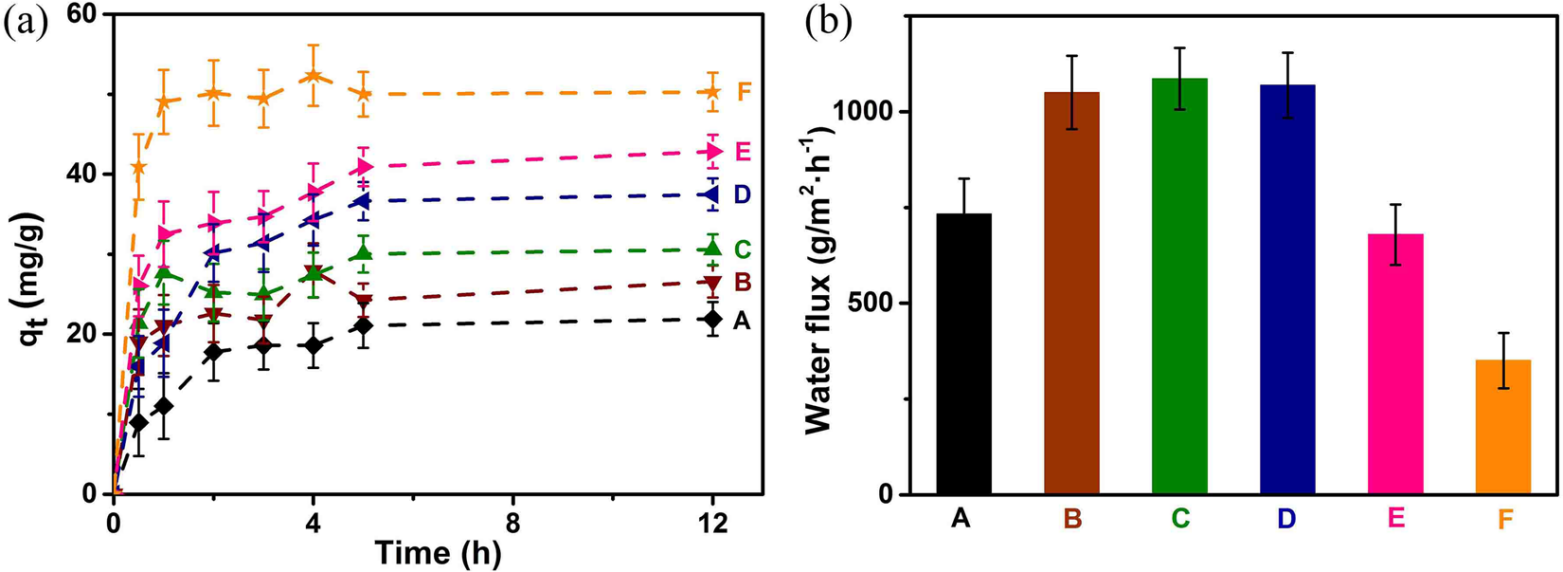
(**a**) Copper adsorption capacity of modified PAN membrane; (**b**) The water flux measurement of modified PAN membrane. (A: Pure PAN membrane, B: PAN-(DR-EDTA)_1_, C: PAN-(DR-EDTA)_2_, D: PAN-(DR-EDTA)_3_, E: PAN-(DR-EDTA)_4_, F: PAN-(DR-EDTA)_5_).

In order to evaluate the adsorption capacity of the prepared membranes, fiber PAN membrane with different modification layers was investigated. As illustrated in Fig. 3a, the copper uptake of the modified PAN membranes increased from 21.9 mg/g to 50.3 mg/g within five modification layers. This could be attributed to the fact that the introduced EDTA chelating groups could effectively coordinate with the metal ions. Better chelating ability of PAN membranes was achieved with increased DR-EDTA layers. Especially, the copper ion uptake could reach 37.54 mg/g when the modified layer was three, comparable to the similar work^27^. The increase of copper adsorption capacity was attributed to the introduction of DR-EDTA layers due to chelation groups. As a result, adsorption capacity of PAN membrane increased with increased DR-EDTA layers.

The water flux of modified PAN membrane was measured (under an operating pressure of 19.79 KPa) and was shown in Fig. 3b. The water fluxes of PAN-(DR-EDTA)_0_, PAN-(DR-EDTA)_1_, PAN-(DR-EDTA)_2_, PAN-(DR-EDTA)_3_, PAN-(DR-EDTA)_4_ and PAN-(DR-EDTA)_5_ fiber membrane were 733.30, 1049.80, 1085.94, 1068.85, 679.30, 350.30 g·m^−1^·h^−1^, respectively. Especially, the water fluxes of PAN-(DR-EDTA)_1_, PAN-(DR-EDTA)_2_ and PAN-(DR-EDTA)_3_ exhibited 143%, 148% and 146% higher than that of the pure fiber membrane, respectively. The improved water flux was mainly attributed to the increased hydrophilic groups in DR and EDTA. The water flux of PAN membrane reached a relatively stable value within three DR-EDTA layers. This phenomenon was due to the comprehensive results originating from the introduced hydrophilic groups in DR-EDTA layer and the blockage on the porous PAN fiber. However, the water flux of PAN sharply declined with the DR-EDTA layer increased above four. Because the latter factor gradually dominates role, inhibiting the water penetration through the modified PAN fiber membrane. Combining the adsorption capacity and the water flux for the modified PAN fiber membrane, the optimized PAN-(DR-EDTA)_3_ fiber membrane was selected as the investigated object below.

**Figure 4:**
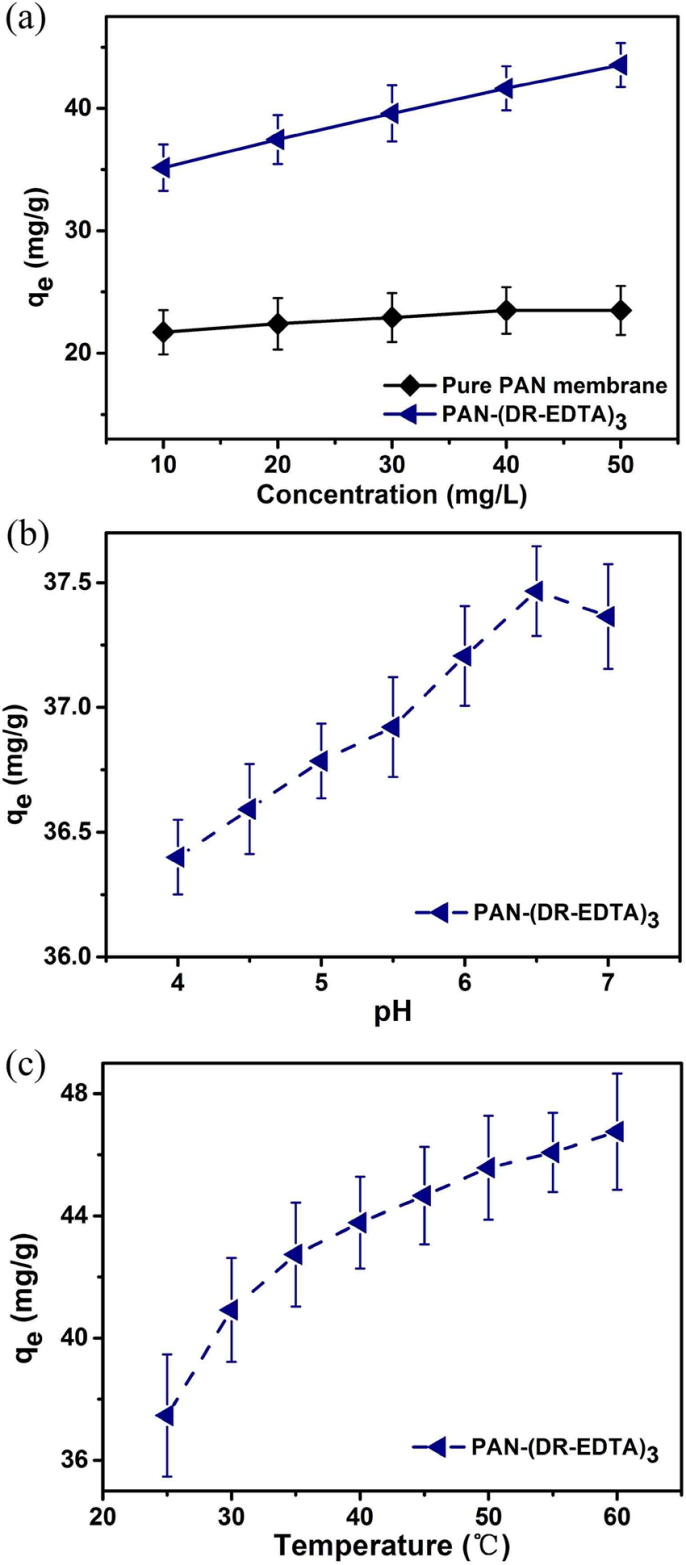
(**a**) Effect of concentration on adsorption capacity, (pH: 6.5, temperature: 25 °C and adsorption time: 720 min) (**b**) Effect of pH on adsorption capacity (Cu^2+^ concentration: 20 mg/L, temperature: 25 °C and adsorption time: 720 min) and (**c**) Effect of temperature on adsorption capacity. (Cu^2+^ concentration: 20 mg/L, pH:6.5, and adsorption time: 720 min).

Dependence of copper adsorption on concentration of adsorbent was carried out under the condition (pH = 6.5, temperature = 25 °C and adsorption time = 720 min). Figure 4a presented the copper adsorption capacity for pure PAN membrane and PAN-(DR-EDTA)_3_. the amount adsorbed of pure PAN membrane increased from 21.7 mg/g to 23.5 mg/g when the initial concentration of copper changed from 10 mg/L to 50 mg/L. The PAN-(DR-EDTA)_3_ was increased from 35.16 mg/g to 43.54 mg/g. It could be observed that the adsorption capacity of pure PAN membrane did not increased too high. For the PAN-(DR-EDTA)_3_, the adsorption capacity varied much with increase of initial concentration of copper. The increase was attributed to the resistance of the copper ions absorption decrease with the increase of the inmetal ions concentration.

Adsorption isotherm models such as Langmuir and Freundlich^31^ were used to analyze the experimental data of the sorption of Cu^2+^ onto PAN membranes. The Langmuir model:

1 $${q}_{e}=\frac{{q}_{m}{k}_{L}{c}_{e}}{1+{k}_{1}{c}_{e}}$$

It could be expressed by:

2 $$\frac{{c}_{e}}{{q}_{e}}=\frac{1}{{k}_{1}{q}_{\max }}+\frac{{c}_{e}}{{q}_{m}}$$

where *c*_*e*_ (mg/L) was the equilibrium concentration; *q*_*e*_ (mg/g) was the equilibrium adsorption capacity; *q*_*m*_ (mg/g) was the maximum adsorption capacity; *k*_1_ (L/mg) was the constant related to the free energy of adsorption. The Freundlich model:

3 $${q}_{e}={k}_{2}{({c}_{e})}^{1/n}$$

where *k*_2_ was Freundlich constants related to the adsorption capacity and *n* was Freundlich constants related to the adsorption intensity.

The Langmuir and Freundlich parameters (Supplemental Fig. S1) were calculated from the the slopes and intercepts of the lines and the data was shown in Table 1. It was clear that the data for the adsorption of Cu^2+^ on to the PAN membrane were well fitted to the Langmuir model. The Freundlich constant (1/n) was smaller than 1 which indicates a favorable process^32^. The *q*_*m*_ calculated from the Langmuir model were 47.6 mg/L for Cu^2+^. The adsorption capacities of other sorbents were shown in Table 2. The comparative results showed that the PAN membrane had higher absorbency.

**Table 1:** Parameters for the Langmuir and Freundlich models of Cu^2+^ adsorption.

Metal	Langmuir		Freundlich	
	*q*_*m*_	K_1_ (L/mg)	K_2_ (mg/g(L/mg)^1/n^)	1/n
Cu^2+^	47.6	0.233	25.70	0.13

**Table 2:** Comparison of the maximum adsorption capacity of Cu^2+^ on various adsorbents.

References	Adsorbent	*q*_*m*_ (mg/g)	Conditions
^40^	MWCNT	24.5	pH 5.0 T = 298 K
^41^	Graphene oxide	46.6	pH 5.0 T = 298 K
^42^	Go-PAMAM	38.4	pH 5.6 T = 298 K
^43^	Go aerogel	19.7	pH 6.3 T = 298 K
^44^	Go-CS aerogel	25.4	pH 6.0 T = 303 K
^45^	CEMNPS	3.2	pH 9.0 T = 298 K
^12^	PD/GO composites	24.4	pH 6.0 T = 298 K
^46^	S-MWCNTS	43.2	pH 5.0 T = 298 K
**This study**	**PAN-DR-EDTA**	**47.6**	**pH 6.0 T = 298 K**

To investigated the copper adsorption capacity of the PAN fiber membrane in harsh environment, the influence of pH and temperature for the PAN-(DR-EDTA)_3_ were studied. As illustrated in Fig. 4b, the copper adsorption capacity of PAN-(DR-EDTA)_3_ increased from 36.3 mg/g to 37.4 mg/g when the pH changed from 4.0 to 6.5. This could be owning to the fact that the acid effect became weaker at higher pH, leading to the increase of conditional stability constant for the EDTA-Copper system. Nevertheless, the hydrolytic reactions would occur for the copper ion at higher pH (>7.0), through which the copper ion compound (Cu(OH)_2_) precipitate out from the waste water^33,34^. Moreover, the nitrogen-containing group (−NR_3_/−NR_2_H) in DR under the acid environment would easily be protonated, and could repel the metal ions penetration through the fiber membrane via the Donnan effect. Based on these considerations, the copper uptake of the PAN-(DR-EDTA)_3_ membrane obtain a much lower adsorption capacity toward copper ion in comparison with the value under the alkaline environment. Because of the mixed coordination effect for the EDTA-Copper and the acid effect for the metal ion^26^, the PAN-(DR-EDTA)_3_ fiber membrane possess a maximum copper ion uptake capacity of 37.4 mg/g when the pH was 6.5.

As shown in Fig. 4c, the copper adsorption capacity of the PAN-(DR-EDTA)_3_ fiber membrane increased with temperature (at pH = 6.5). The free volume of polymer would increase with temperature, further improving copper ion uptake of polymer^32,35,36^. Especially, the copper ion uptake of PAN-(DR-EDTA)_3_ could reach 46.2 mg/g at 65 °C, which increased by 24% in comparison with 37.4 mg/g of the fiber membrane at 23 °C. The obtained results suggested that the polymer adsorption for copper ions can be regulated with the temperature, showing good application flexibility.

**Figure 5:**
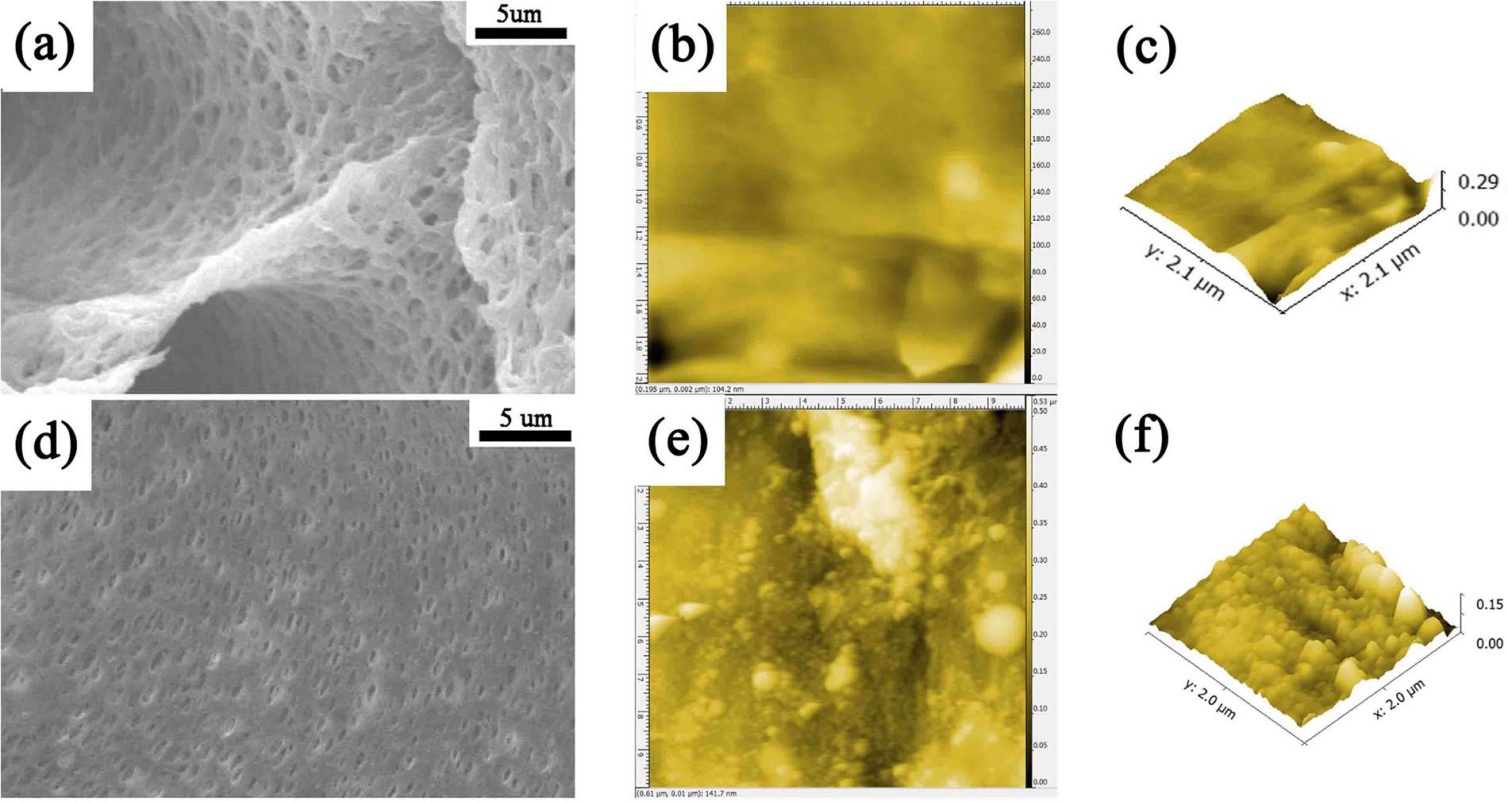
(**a**,**d**) SEM images of pure PAN membrane and PAN-(DR-EDTA)_3_ membrane, showing an internal construction of PAN membrane. (**b**), (**c**) and (**e**,**f**) AFM images of pure PAN membrane and PAN-(DR-EDTA)_3_ membrane, showing a surface shapes of PAN membrane.

The morphology and topography of the pure PAN and PAN-(DR-EDTA)_3_ fiber membrane was measured by SEM and AFM (Fig. 5). As could be seen in Fig. 5a and d, similar with the pure PAN membrane, a typical symmetric sponge-like PAN-(DR-EDTA)_3_ fiber membrane was observed, with interconnected porous structure. However, the pores density of the PAN-(DR-EDTA)_3_ was higher than that of the pure PAN membrane due to the introduced DR-EDTA layers^37^. In addition, the hydrophilicity of the PAN fiber membrane would increase mainly due to the introduced –COOH groups in EDTA. As a result, the water flux of the modified PAN fiber membrane changed caused by these two contradictory factors, consistent with the results in Fig. 3b. The surface morphology of PAN-(DR-EDTA)_3_ was more rough than the pure PAN membrane (Fig. 5b,e). After calculation on the AFM surface of the fiber membrane, the average interface roughness (Ra) value for the pure PAN and the PAN-(DR-EDTA)_3_ fiber membrane were 15.9 nm and 57.6 nm, respectively (Fig. 5c,f). These results further confirmed that the DR-EDTA layer was successfully grafted on the pure PAN fiber membrane. More importantly, the increased roughness of the modified PAN membrane could increase the contact area between the copper ion/water and the chelating groups in DR-EDTA. And therefore, enhanced copper ion/water uptake of the PAN membrane was obtained. The SEM images of membrane with different DR-EDTA layer coating was shown in Supplemental Fig. S2. The pores in the membrane became smaller with the number of DR-EDTA layers increased.

To investigate the surface wettability of the PAN membrane, the hydrophilicity of the membrane with different layers of DR-EDTA has been studied. As shown in Supplemental Figure S3, the introduced DR-EDTA layer could remarkably improve the hydrophilicity of the pure PAN fiber membranes, and then greatly enhance the PAN fiber membrane water flux.

**Figure 6:**
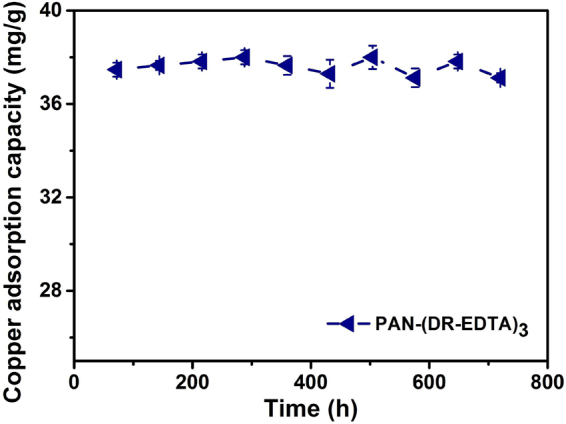
Adsorption stability of PAN-(DR-EDTA)_3_ over 720 hours.

To investigated the stability of the modified PAN membrane, the fiber membrane adsorption/desorption experiments toward copper ions were measured for 720 h. Figure 6 showed the copper adsorption capacity during the testing process. It showed that the PAN-(DR-EDTA)_3_ membrane still maintains a strong adsorption capacity for copper ions (37.12 mg/g) after 10 times adsorption-desorption test, in comparison with its initial value (37.47 mg/g). These results proved that the PAN-(DR-EDTA)_3_ membrane possess stable absorption/desorption ability for copper ions, implying the good reusability of the as-prepared fiber membrane.

The thermogravimetric analysis (TGA) of the pure PAN and PAN-(DR-EDTA)_3_ fiber membrane was shown in Supplemental Fig. S4. Compared pure fiber PAN membrane, thermal stability of PAN-(DR-EDTA)_3_ changed.

Except for the superior adsorption ability toward copper ion, the adsorption ability of the PAN-(DR-EDTA)_3_ fiber membrane for other heavy metal ions (Cd^2+^, Hg^2+^, Pb^2+^) were also investigated (Supplemental Fig. S5). The PAN-(DR-EDTA)_3_ membrane could simultaneously absorb the different metal ion. This could be attributed to the different coordination ability between the grafted EDTA and the metal ion.

## Methods

### Materials

PAN membrane were purchased from Shandong Jinhui film Polytron Technologies Inc. Diazo-resin (DR, *Mn* = 2500) was synthesized according to a method reported elsewhere^38^. EDTA, cresol red, ammonia chloride and sodium diethyldithiocarbamate trihydrate were all obtained from Sinopharm group Co. ltd.

### Characterization

Scanning electron microscopy (SEM, JEOL JSM-6309LV), operating at a 20 kV accelerating voltage, was used to characterize the inner structure of PAN membrane and the surface characterization of the coatings was characterized by atomic force microscope (AFM, CSPM 5500, China). FTIR spectra of PAN membrane was measured in Bruker alpha type infrared spectrometer. Thermogravimetric curves of PAN membrane and modified membrane were obtained on SDTQ600 comprehensive thermal analyzer. Contact angles of PAN membrane were characterized with an automated contact angle goniometer (Dingsheng JY-82), which was recorded at 20 s after a water drop to get a steady reading. APuxi TU-1810 UV-Vis spectrometer was detected for UV-vis absorbance of copper solution.Citrate buffer solution was used as acidity regulators.

### Pretreatment of PAN membrane

The PAN membrane was cut into several parts and each paragraph was four to five centimeter. The parts were hydrolyzed by immerging in NaOH solution (1 M) for 1 h and washed by distilled water until the pH of solution was 7. Finally, the parts were dried in vacuum.

### Preparation of the DR-EDTA-coated membranes

The hydrolyzed PAN membranes were immersed in DR solution (1 mg/mL) for 24 h. Then the membranes were immersed into distilled water for 5 times to remove extra DR. Similarly, the above membranes were treated with EDTA solution (1.5 mg/mL) for 24 h and washed by distilled water and dried in vacuum. Finally, the photo-crosslinking of the DR/EDTA coating on the membranes were carried out. The scheme of grafting DR and EDTA onto the PAN membrane surface was shown in Fig. 1.

### Preparation of the multilayer DR-EDTA-coated membranes

The above PAN-(DR-EDTA)1 membranes were immersed into distilled water. DR and EDTA solutions were taken in turns to deal with PAN membrane. And then the membrane was exposed under UV curing system alternately to obtain the multilayer DR-EDTA-coated membranes.

### The metal ions adsorption

The adsorption measurements of the modified PAN membranes were carried out by UV-Vis spectrophotometry according to the refercences reported. Adsorption experiments were carried out in a series of beakers of 50 mL. The beakers contained expected amount of heavy metal ion solution and were stirred on the heating magnetic stirrer. The effects of contact time (0–720 min), concentration (10, 20, 30 and 40 mg/L), solution pH (4, 4.5, 5, 5.5, 6 6.5 and 7) and temperature (25 °C, 30 °C, 35 °C, 40 °C, 45 °C, 50 °C, 55 °C and 60 °C) were investigated. The pH value was adjusted using hydrochloric acid sodium hydroxide. After required adsorption time, the solution was dealt with sodium diethyldithiocarbamate trihydrate. The copper concentration could be analysed by using a UV–vis spectrophotometer after extraction. The adsorption capacity was calculated using^31^:

4 $${q}_{t}=\frac{({c}_{0}-{c}_{t})V}{M}$$

where the *C*_0_(mg/L) and *C*_1_(mg/L) were the copper ion concentration before and after adsorption, respectively, *V*(L) was the volume of aqueous solution and M(g) was the weight of the adsorbent.

## Conclusions

In summary, an advanced PAN-based membrane with enhanced adsorption property for heavy metal ions was prepared by layer-by-layer self-assembly method. The introduced DR-EDTA layer could effectively absorb the metal ion in the waste water. The adsorption property of the polyacrylonitrile-based membrane could be controlled by adjusting the modification layers. The maximum adsorption capacity of PAN-(DR-EDTA)_3_ toward Cu^2+^ was approximately 47.6 mg/g. In addition, the PAN-(DR-EDTA)_3_ membrane exhibit superior reusability after 720 h adsorption-desorption test, showing a very promising prospect for wastewater treatment^39^.

## Electronic supplementary material

Supplementary information
