# Supplementary material for: Advanced Modified Polyacrylonitrile Membrane with Enhanced Adsorption Property for Heavy Metal Ions
Source: Sci Rep. 2018 Jan 19;8:1260. doi: 10.1038/s41598-018-19597-3 (PMC5775326; doi:10.1038/s41598-018-19597-3)
Supplement: Supplementary file 1 — Supplementary information [file 41598_2018_19597_MOESM1_ESM.pdf]

Advanced Modified Polyacrylonitrile Membrane with Enhanced Adsorption Property for Heavy Metal Ions

Xinfeng Zhang[1], Shujing Yang[1], Bing Yu[1,2], Qinglong Tan[1], Xiaoyan Zhang[1], Hailin Cong[1,2*]

[1]Institute of Biomedical Materials and Engineering, College of Chemistry and Chemical Engineering, Qingdao University, Qingdao 266071, China

[2]Laboratory for New Fiber Materials and Modern Textile, Growing Base for State Key Laboratory, College of Materials Science and Engineering, Qingdao University, Qingdao 266071, China

[*]hailincong@yahoo.com

Supplementary data file

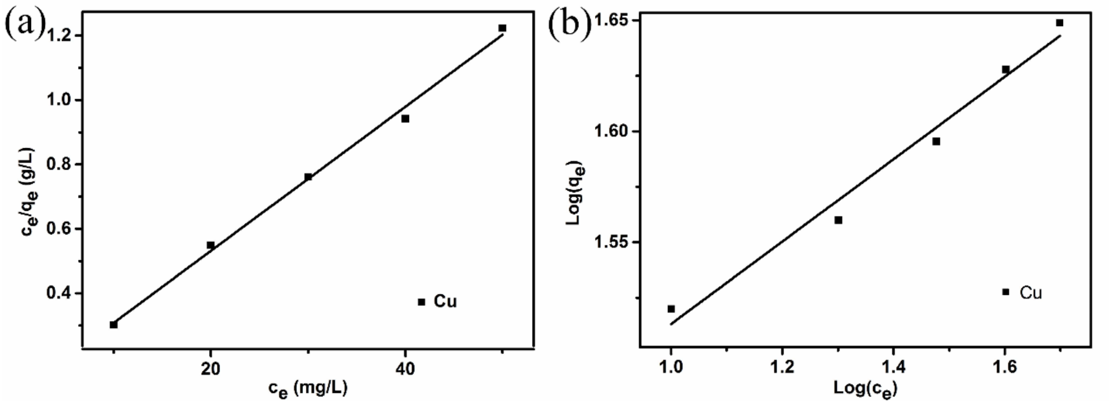

Supplemental Figure S1. Langmuir (a) and Freundlich (b) isotherms for the adsorption of Cu^{2+} onto modified PAN membranes. (T = 303 K, pH 6.0, adsorption time=720 min).

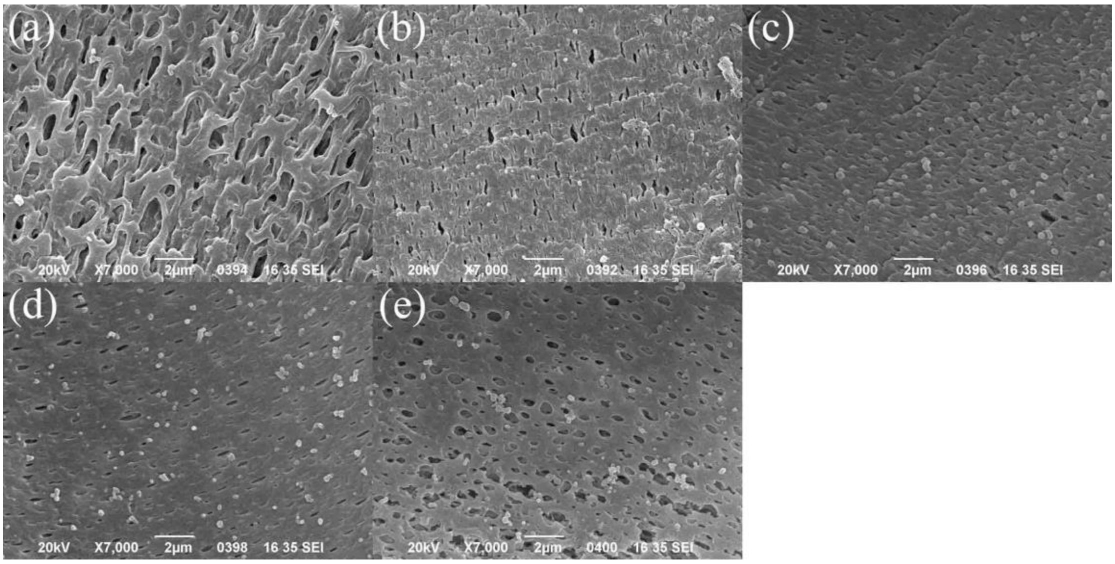

Supplemental Figure S2. SEM of (a)PAN-$(DR\text{-}EDTA)_1$, (b)PAN-$(DR\text{-}EDTA)_2$, (c)PAN-$(DR\text{-}EDTA)_3$, (d)PAN-$(DR\text{-}EDTA)_4$, (e)PAN-$(DR\text{-}EDTA)_5$.

Supplemental Fig. S2 showed the SEM images of membrane with different DR-EDTA layer coating. The SEM provided the direct observation of changes in the surface due to the increase of DR-EDTA layers. The pores in the membrane became smaller with the number of DR-EDTA layers increased. The collapse occurred when the number of layers increased to five.

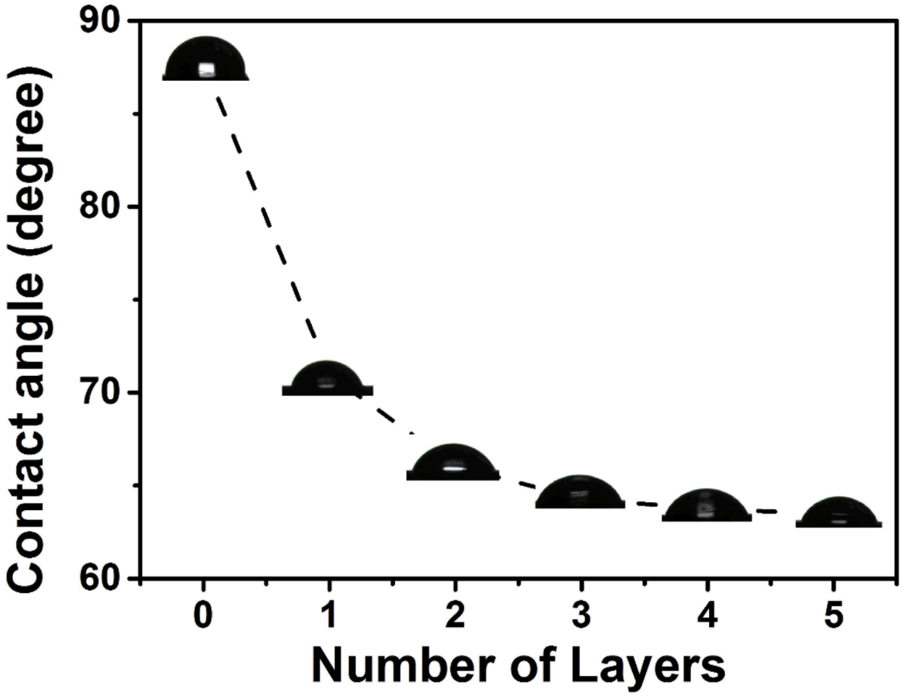

Supplemental Figure S3. Water contact angle of pure PAN membrane and modified PAN membrane.

The hydrophilicity of the modified PAN membrane was studied by the water contact angle measurement. Every membrane we measured was cut from the side of the membrane. And then the film was sandwiched between two smooth glass sheets with a heavy weight of 700 g pressure for 24 hours. Finally, the obtained flat membrane was used to execute the water contact angle test. As demonstrated in Supplemental Fig. S3, the water contact angle of modified PAN membrane decreases from 87.8^{o} to 64.3 $^{\circ}$ within five DR-EDTA layers. These results suggested that the introduced DR-EDTA layer could remarkably improve the hydrophilicity of the pure PAN fiber membranes, and then greatly enhance the PAN fiber membrane water flux.

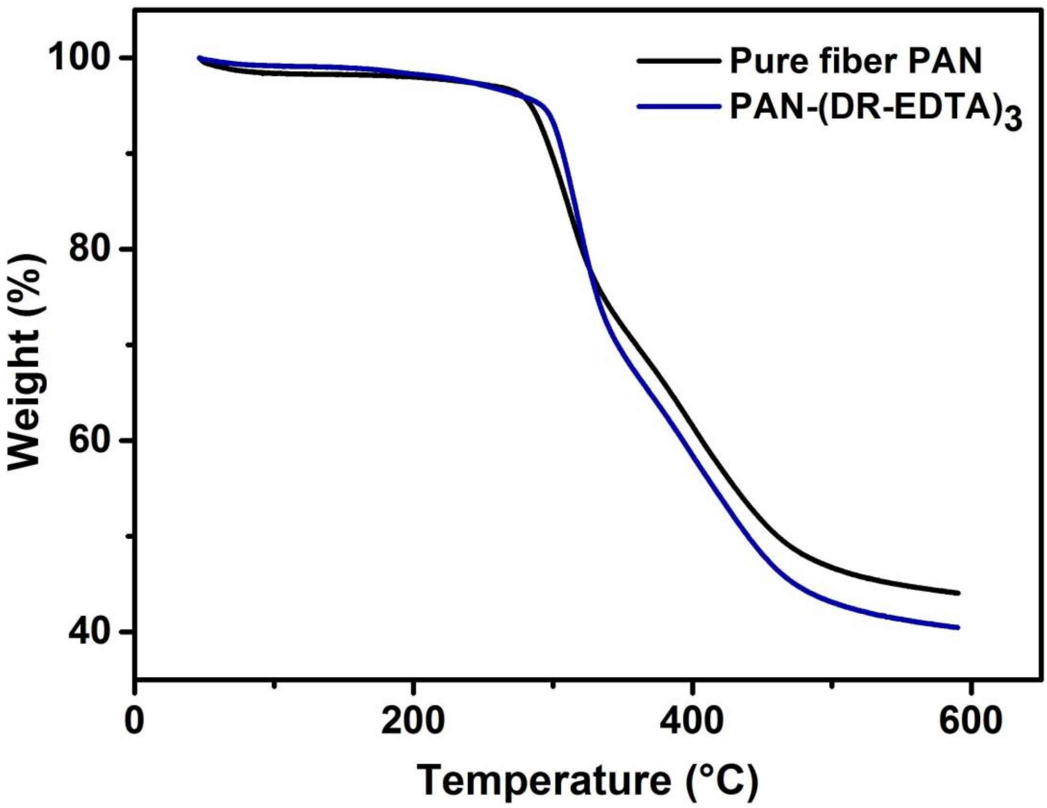

Supplemental Figure S4. TGA curves for pure fiber PAN and PAN-(DR-EDTA)$_3$ membrane.

The thermogravimetric analysis (TGA) of the pure PAN and PAN-(DR-EDTA)$_3$ fiber membrane was shown in Supplemental Fig. S4. It could be seen that the PAN-(DR-EDTA)$_3$ had a similar weight loss compared with the pure PAN fiber membrane below 600 ℃, indicating an excellent thermal stability. The degradation of PAN-(DR-EDTA)$_3$ fiber membrane could be divided into three stages. The first stage (25-278 ℃) could be attributed to the water evaporation. The second stage (278-550 ℃) could be ascribed to pure PAN, DR and EDTA chain scission, including the produced HCN, CO and CO_2 gases. The third stage (550-600 ℃) was owning to the degradation of carbonaceous matters of the polymer.

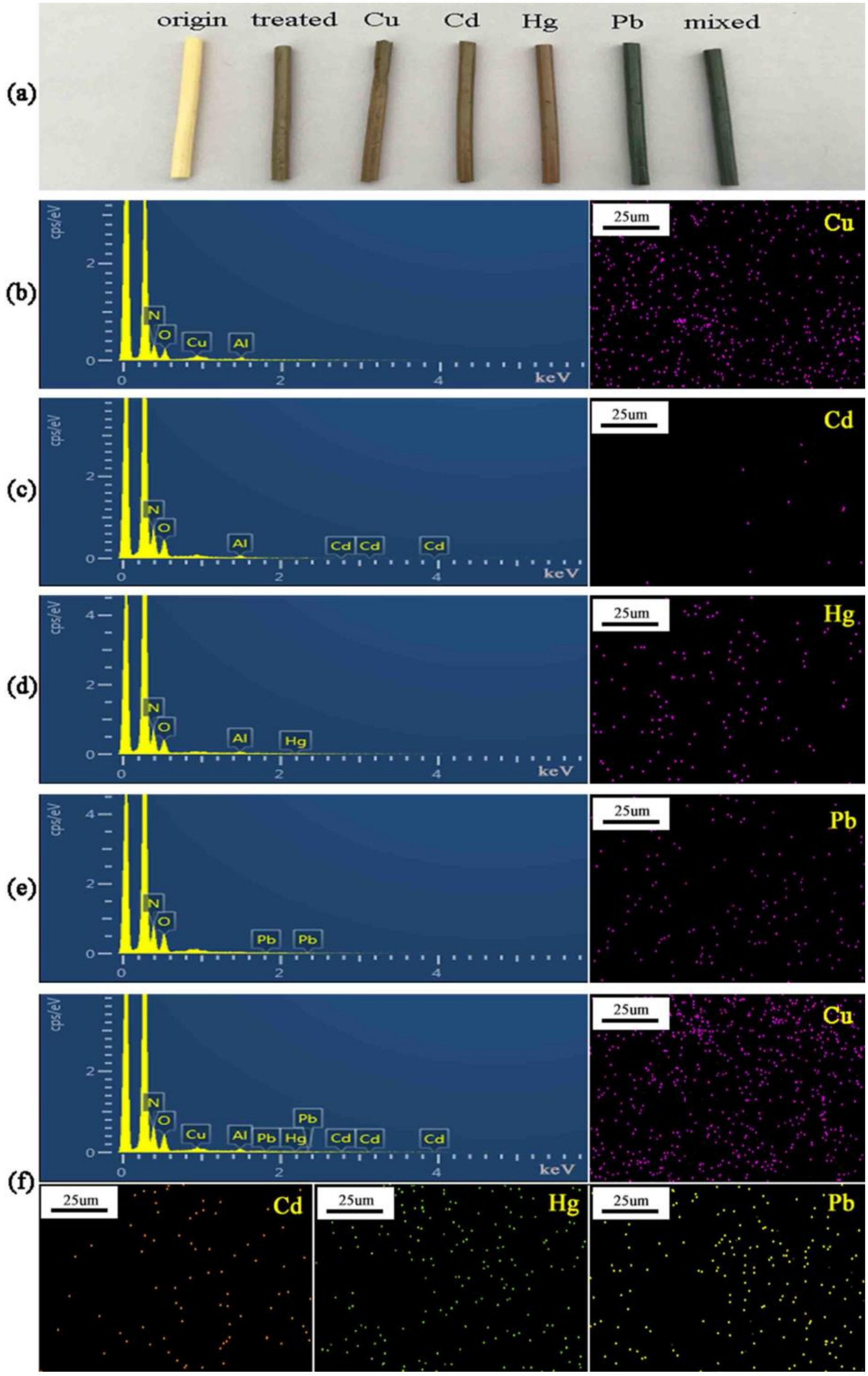

Supplemental Figure S5. (a) Image of PAN membrane of absorbing different heavy metal ion, (b)~(e) EDS of PAN membrane of absorbing different heavy metal ion, (f) EDS of PAN membrane of absorbing mixed solution.

Except for the superior adsorption ability toward copper ion, the adsorption ability of

the PAN-(DR-EDTA)$_3$ fiber membrane for other heavy metal ions (Cd^{2+}, Hg^{2+}, Pb^{2+}) were also investigated. As shown in Supplemental Fig. S5a, the color change for the PAN-(DR-EDTA)$_3$ fiber membranes suggested the membrane adsorption ability toward Cu^{2+}, Cd^{2+}, Hg^{2+} and Pb^{2+}. And the EDS mapping of the PAN-(DR-EDTA)$_3$ further confirm the fact (Supplemental Fig. S5b-Fig. S5e). Supplemental Fig. 6f showed the PAN-(DR-EDTA)$_3$ absorption for the mixed solution including the Cu^{2+}, Cd^{2+}, Hg^{2+} and Pb^{2+}, exhibiting good absorption property. The result suggested that the PAN-(DR-EDTA)$_3$ membrane could simultaneously absorb the different metal ion. This could be attributed to the different coordination ability between the grafted EDTA and the metal ion.
